# Extraction, Physicochemical Properties, Anti-Aging, and Antioxidant Activities of Polysaccharides from Industrial Hemp Residues

**DOI:** 10.3390/molecules27185746

**Published:** 2022-09-06

**Authors:** Tanran Chang, Hang Li, Hongning Lv, Minghui Tan, Shoubu Hou, Xu Liu, Meng Lian, Qingsheng Zhao, Bing Zhao

**Affiliations:** 1 State Key Laboratory of Biochemical Engineering, Institute of Process Engineering, Chinese Academy of Sciences, Beijing 100190, China; 2 University of Chinese Academy of Sciences, Beijing 100049, China; 3 College of Food Science and Technology, Guangdong Ocean University, Zhanjiang 524088, China; 4 Yunnan Hempmon Pharmaceuticals Co., Ltd., Kunming 650032, China

**Keywords:** industrial hemp, polysaccharide, extraction, antioxidant activity, anti-aging activity

## Abstract

A large amount of hemp polysaccharides remain in industrial hemp residues (IHR) after cannabidiol extraction, resulting in the waste of resources. Therefore, the systematic study of hemp polysaccharides is beneficial to the development of IHR in the future. In this study, the extraction of industrial hemp residues polysaccharide (IHRPs) was optimized by single-factor experiment and orthogonal experimental design. The optimum heating extraction conditions were extraction temperature 98 °C, solid–liquid ratio 1:10, extraction time 1 h, number of successive extractions 2, and pH at 4. The extraction ratio and the polysaccharide content were 20.12 ± 0.55% and 12.35 ± 0.26% at the conditions, respectively. Besides, the best alcohol precipitation conditions were pumping with 2 L/h, stirring continuously, and ice-water bath for 4 h. The crude IHRPs was further purified by column chromatography and the polysaccharide/protein contents of purified IHRPs were 34.44% and 1.61%. IHRPs was mainly made up of ten monosaccharides and some non-sugar components including organic acids, flavonoids, steroids, and glycoside. The FT-IR demonstrated the polysaccharide skeleton of IHRPs. Moreover, the DPPH and ABTS scavenging rate of IHRPs were 76.00% and 99.05% at the concentrations of 1 mg/mL. IHRPs could promote the epidermal cells proliferation and healing of cell scratches. Meanwhile, IHRPs could promoted the expression of anti-aging-related genes. Overall, IHRPs could be a desirable natural source of antioxidants and anti-aging products in many aspects.

## 1. Introduction

Hemp (*Cannabis sativa* L.), an annual herb of the *Cannabis* family, has been used for thousands of years [1]. The tetrahydrocannabinol (THC) content of industrial hemp is less than 0.3 wt%, which is different from medical marijuana and recreational marijuana [2,3]. Therefore, industrial hemp could be legally cultivated on a large scale. The chemical composition of industrial hemp is very complex, including cannabinoids, terpenoids, alkaloids, and flavonoids [4]. The most bioactive compounds are cannabinoids that include more than 130 terpene phenolic compounds which mainly accumulate in the flower tops [5]. The major cannabinoids are THC, cannabidiol (CBD), cannabinol (CBN), cannabigerol (CBG), cannabidivarin (CBDV), cannabitriol (CBT), and cannabicyclol (CBL) [6]. Among them, THC, a psychoactive compound restricted in different countries, and CBD are the most studied cannabinoids for their significant bioactivities [7], such as neuroprotective, antiepileptic, anticancer, and immunoregulatory activities [8]. The existence of these compounds increases the importance of industrial hemp. At present, industrial hemp has been widely used in the fields of daily chemicals, materials, energy and medicine [9]. The current treatment of industrial hemp residues (IHR) is directly discarding them, which not only pollutes the environment, but also causes a waste of resources [10].

Polysaccharide is a polymer which consists of exceeding 10 monosaccharides [11]. It is one of the most essential biological macromolecules. In recent years, many articles have reported that the natural polysaccharides from plants have good biological activities, including anticancer [12], anti-inflammatory [13], antioxidant [14], immunomodulatory [15], hypoglycemic [16], and antiviral activities [17]. The molecular composition and spatial structure of polysaccharides generally have significant effects on their properties and activities [18]. With the development of separation, purification, and structural analysis technology of polysaccharide, it is generally accepted that polysaccharides have great application values in drug, cosmetics, and food fields [19]. The content of polysaccharides from industrial hemp residues is about 10%–15%. Hemp polysaccharides have many excellent physicochemical properties because they contain lots of hydrophilic hydroxyls, such as strong water-absorption capability, emulsifying properties, and good film-forming ability. Meanwhile, hemp polysaccharides have moisture retention, antibacterial, and no-toxic effects. Therefore, they have extensive application prospects in the cosmetics field. The fermented products of hemp polysaccharides (oligosaccharides or some sugar alcohols), which are reported to have good antioxidant, anti-aging, and anti-fatigue activities, have been used to develop functional drinks [20]. The composition of hemp polysaccharides is relatively complex and it ranges from monosaccharides to 5 × 10^4^ Da polysaccharides.

There are few reports about the separation and extraction of hemp polysaccharides. Hillestad et al. isolated two glycoproteins from hemp. The linkage structure of glucoside chain and the connection between the chain and protein were analyzed by methylation and Smith degradation [21]. J.W. Groce et al. measured the carbohydrate content of hemp grown in the United States, Thailand, and Vietnam respectively. It was pointed out that large differences existed in the type and content of compounds between the cultivars from different areas [22]. Zheng et al. analyzed the monosaccharide composition and structure of HSP0 and HSP0.2, two components of hemp seed polysaccharide, by HPLC and FT-IR. It was found that HSP0 and HSP0.2 both contained sulfate radical [23]. Bi et al. obtained two hemp seed polysaccharides HS1 and HS2 by gel filtration chromatography. The main monosaccharides were D-arabinose, D-xylose, D-mannose, D-galactose, and D-glucose, and their molar ratios were 0.12, 0.09, 0.15, 0.11, and 0.12, respectively [24]. Guo et al. prepared polysaccharides from industrial hemp leaves and studied the effects of ultraviolet light, temperature, and pH on their antibacterial activity. It was found that the minimum bactericidal concentration and minimum inhibition concentration of the polysaccharides against *S. aureus* were 6.25 and 3.125 mg/mL, respectively. Additionally, the antibacterial activity was the highest when pH was 7.

In this study, the IHR after cannabidiol extraction were used as the raw material for polysaccharides extraction. The extraction and purification process of industrial hemp residue polysaccharides (IHRPs) were optimized. Then the structure and composition of IHRPs were analyzed. Besides, the ABTS and DPPH scavenging ability of IHRPs was evaluated. Its anti-aging activity was also studied at the cellular level. It is hoped that IHRPs could be widely used in cosmetics and functional food fields.

## 2. Results and Discussion

### 2.1. Single Factor Experiments of Extraction for IHRPs

#### 2.1.1. Effects of Extraction Methods on Polysaccharide Extraction

**Figure 1:**
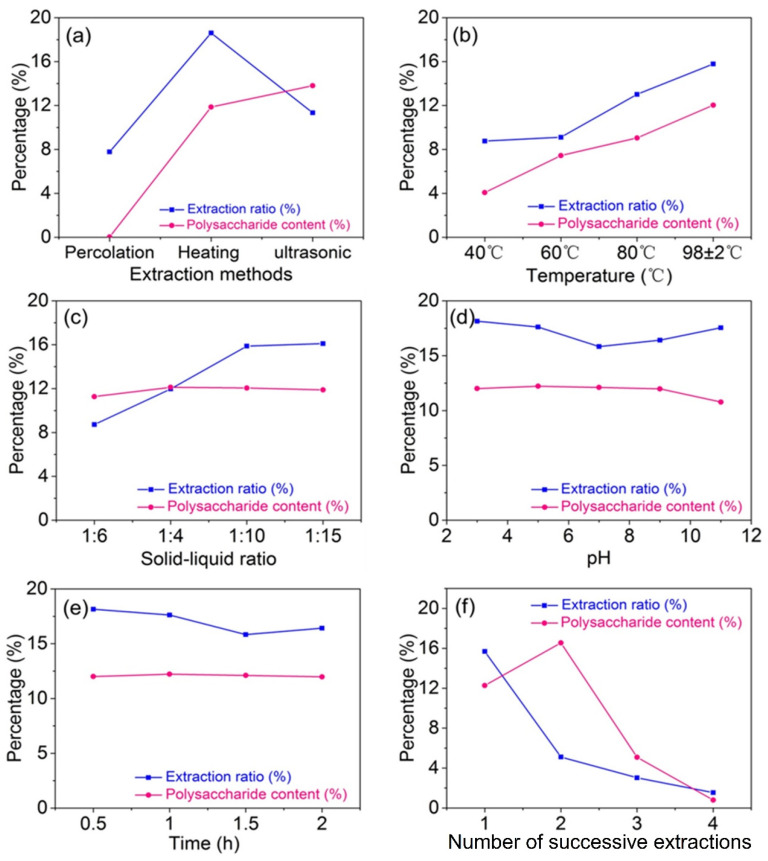
Effects of (**a**) extraction methods, (**b**) temperatures, (**c**) solid–liquid ratios, (**d**) pH, (**e**) extraction time, and (**f**) number of successive extractions on polysaccharide extraction.

The effects of percolation extraction, heating extraction, and ultrasonic-assisted extraction on polysaccharide extraction are shown in Figure 1a. It could be seen that the polysaccharide content of percolation extraction was close to 0%, which indicated that nearly no polysaccharide was extracted and the extract mainly consisted of pigment and other water-soluble components. The results were due to the percolation extraction being very gentle and the cell wall of IHR not being broken. Thus, the polysaccharides could not be extracted from cells, which caused the polysaccharide content and extraction ratio of percolation extraction to be very low. The extraction ratio of heating extraction was higher than that of ultrasonic-assisted extraction. However, the polysaccharide content of heating extraction (11.27%) was lower than that of ultrasonic-assisted extraction (13.81%). Heating extraction is one of the most commonly used extraction methods because of the process simplicity and low cost. However, the time of heating extraction is relatively long and the extraction ratio is not high. Extraction temperature generally has a great influence on the extraction ratio. The higher extraction ratio of heating extraction in this work was because the heating extraction was performed at 98 °C while the ultrasonic-assisted extraction was performed at 60 °C. Ultrasonic-assisted extraction could shorten the extraction time because the cavitation effect and mechanical effect could destroy cell walls, improve the penetration of the solvent, and accelerate the transfer of polysaccharides to the solvent [25]. Thus, the polysaccharide content of ultrasonic-assisted extraction was higher than that of heating extraction. The heating extraction was chosen for the following experiments in view of process simplicity and industrialization.

#### 2.1.2. Effects of Extraction Temperature on Polysaccharide Extraction

The effects of extraction temperature on polysaccharide extraction were shown in Figure 1b when the solid–liquid ratio was 1:10, extraction time was 1 h, number of successive extractionswas 2, and pH was 7. The extraction ratio and polysaccharide content were both increased with the increase of temperature. When the temperature was 98 °C, the highest extraction ratio and polysaccharide content were 14.99% and 12.03%, respectively.

#### 2.1.3. Effects of Solid–Liquid Ratio on Polysaccharide Extraction

When the extraction temperature was 98 °C, extraction time was 1 h, number of successive extractionswas 2, and pH was 7, the effects of solid–liquid ratio on polysaccharide extraction were displayed in Figure 1c. The extraction ratio increased gradually with solid–liquid ratio from 1:6 to 1:10, and then became smooth. However, the polysaccharide content was nearly not affected by solid–liquid ratio. Besides, the solution could just submerge the raw material when the solid–liquid ratio was 1:5 because of the low density of plant leaves. Therefore, the solid–liquid ratio of 1:6 was studied primarily.

#### 2.1.4. Effects of pH on Polysaccharide Extraction

When the extraction temperature was 98 °C, extraction time was 1 h, number of successive extractions was 2, and solid–liquid ratio was 1:10, the effects of pH on polysaccharide extraction were displayed in Figure 1d. When the solution was acidized or alkalified gradually, the extraction ratio was improved to some extent. However, the polysaccharide content had a decrease trend with the increase of pH, which indicated that the acidic condition was beneficial to the extraction of IHRPs.

#### 2.1.5. Effects of Extraction Time on Polysaccharide Extraction

When the extraction temperature was 98 °C, pH was 7, number of successive extractions was 2, and solid–liquid ratio was 1:10, the effects of extraction time on polysaccharide extraction were displayed in Figure 1e. The extraction ratio decreased lightly when the extraction time exceeded 1 h. Furthermore, the polysaccharide content was nearly not influenced by the different extraction time.

#### 2.1.6. Effects of Number of Successive Extractions on Polysaccharide Extraction

When the extraction temperature was 98 °C, extraction time was 1 h, pH was 7, and solid–liquid ratio was 1:10, the effects of number of successive extractions on polysaccharide extraction were showed in Figure 1f. The extraction ratio dropped to 5.11% when the raw material was extracted the second time. Besides, the polysaccharide content was very low when the raw material was extracted the third time.

### 2.2. Orthogonal Experimental Design of Extraction for IHRPs

It could be seen that extraction temperature, solid–liquid ratio, number of successive extractions, and pH had great influence on polysaccharide extraction based on the single factor experiments. Therefore, the orthogonal experiments were carried out to further optimize the extraction conditions. As shown in Table 1, the order of effect of individual factor on polysaccharide extraction was: extraction temperature > number of successive extractions> solid–liquid ratio > pH. The group 5 (Solid–liquid ratio was 1:8, number of successive extractionswas 3, extraction temperature was 80 °C, and pH was 4) had the highest polysaccharide content (12.79%) while the group 9 (Solid–liquid ratio was 1:10, number of successive extractionswas 2, extraction temperature was 98 °C, and pH was 4) had the second highest polysaccharide content (12.26%). Considering the energy consumption and operability, the comparison validation between the group 5 and 9 was conducted to determine the most appropriate extraction conditions. As shown in Table 2, the verified experiments showed that the polysaccharide content between group 5 and 9 were very close. Thus, to save energy consumption, the extraction conditions of group 9 were selected to extract IHRPs for its use in the following experiments: solid–liquid ratio was 1:10, extraction temperature was 98 °C, number of successive extractions was 2, and pH was 4.

### 2.3. Screen of IHRPs Alcohol Precipitation Conditions

The IHRPs extraction solution was obtained according to the extraction conditions optimized by 2.2. The effects of different alcohol precipitation conditions were displayed in Table 3. Compared with one-time addition and stirring for 0.5 h, pumping and stirring continuously could further improve the polysaccharide content. However, the effect of cooling rate on polysaccharide content was not obvious. Therefore, the alcohol precipitation conditions of group 4 were chosen for the preparation of IHRPs.

### 2.4. Polysaccharide Yield and Chemical Composition of Purified IHRPs

**Figure 2:**
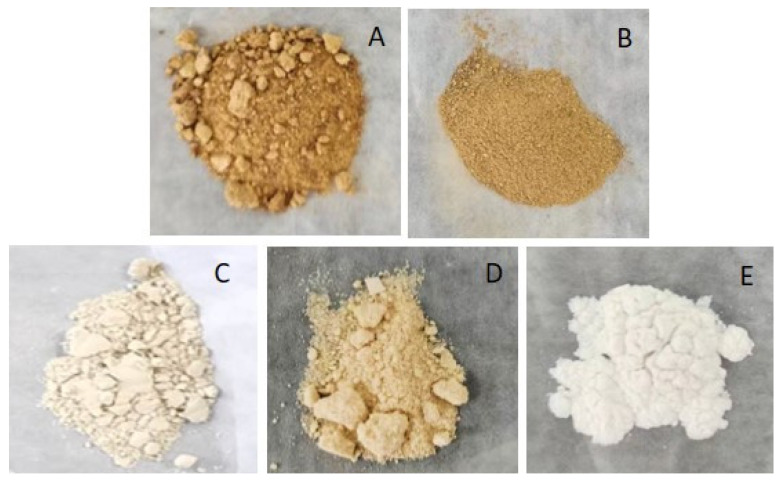
The images of IHRPs purified by different methods: (**A**) sevage method; (**B**) 500 Da membrane filtration; (**C**) column chromatography; (**D**) 4% activated carbon adsorption; (**E**) 8% activated carbon adsorption.

The crude IHRPs were decolorized and purified by activated carbon adsorption, membrane filtration, column chromatography, and sevage method, respectively. As shown in Figure 2, the treatment with 8% activated carbon adsorption had the best decolorizing effect, followed by the column chromatography treatment. Besides, the sevage method and membrane filtration had almost no decolorizing effect. Considering industrialization and simplicity of process, the column chromatography with anion exchange resin was used to purify IHRPs in this manuscript. Besides, as displayed in Table 4, the IHRPs’ weight was 15.26 g when the purification was conducted after the alcohol precipitation, while IHRPs’ weight was 12.74 g when the purification was conducted directly after heating extraction. However, the actual polysaccharide weight of the two processes were very close (4.69 g and 4.38 g). Therefore, to save costs, the column chromatography purification could be carried out directly after heating extraction.

### 2.5. FT-IR Spectroscopy of IHRPs

**Figure 3:**
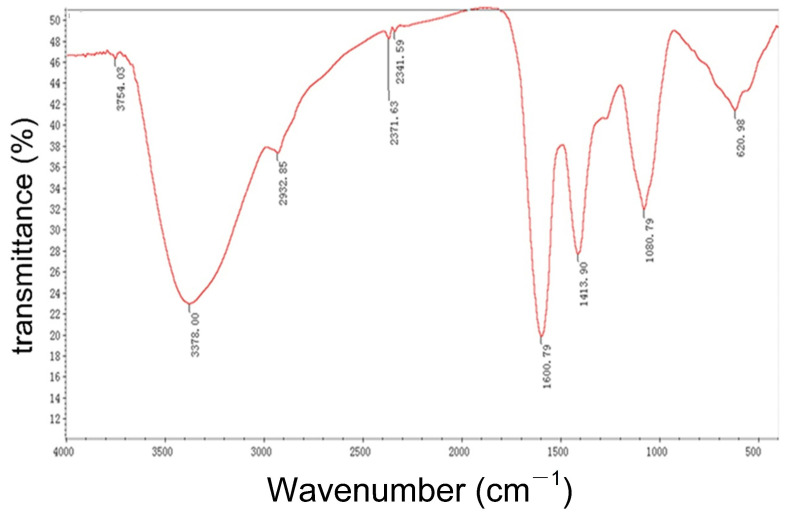
FT-IR spectrum of IHRPs.

Some structural characteristics of polysaccharides could be inferred based on the characteristic absorption peaks of FT-IR [26]. The FT-IR spectrum of IHRPs is shown in Figure 3 and the typical absorption of polysaccharide could be observed. The peak around 3378 cm^−1^ was attributed to -OH stretching vibration, indicating the existence of -OH [27]. The weak peak at 2932 cm^−1^ was C-H stretching vibration, including the vibration of -CH_2_ and -CH_3_ [28]. The strong peak at about 1600 cm−^1^ was related to the -C=O stretching vibration [29], and the peak around 1413 cm^−1^ was denoted for the C-O stretching vibration, indicating the existence of -COOH. Besides, the relatively strong peak at 1080 cm^−1^ very likely corresponds to the O-H bending vibration. The FT-IR spectrum of IHRPs displayed many characteristic absorption peaks of polysaccharides, demonstrating the polysaccharide skeleton of IHRPs.

### 2.6. Monosaccharide Composition Analysis of IHRPs

**Figure 4:**
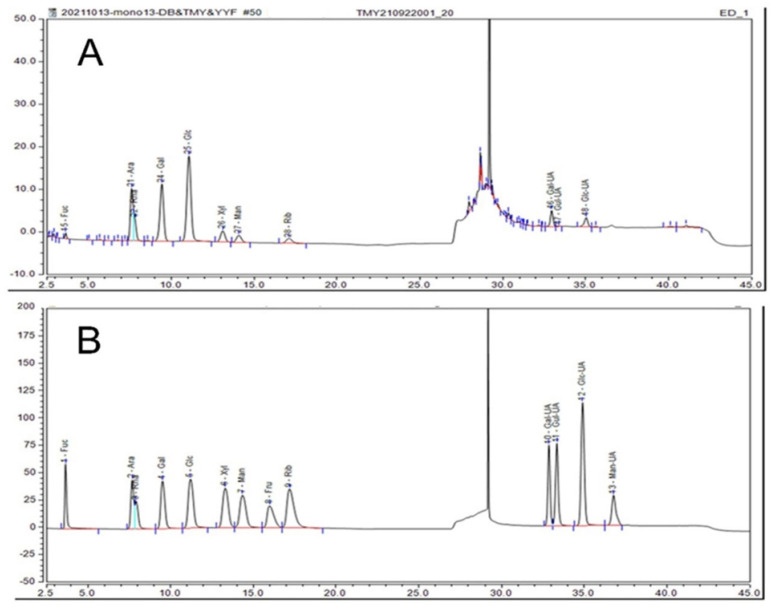
Ion-chromatographic spectra of monosaccharide composition of IHRPs: (**A**) standard monosaccharide and (**B**) IHRPs sample.

The bioactivity of polysaccharide is frequently related to the monosaccharide compositions. In this study, the monosaccharide composition of IHRPs was acquired by ion chromatography as displayed in Figure 4. According to the monosaccharide standards, IHRPs was composed of fucose, arabinose, rhamnose, galactose, glucose, xylose, ribose, galacturonic acid, guluronic acid, and glucuronic acid. Their quality percentages were 1.33, 19.60, 10.41, 20.87, 27.42, 4.23, 3.12, 6.22, 0.28, and 2.37 respectively. It could be found that arabinose, rhamnose, galactose, and glucose were the main monosaccharides in IHRPs. Glucose was a dominant monosaccharide in IHRPs and the result was consistent with the previous study [30]. The content of uronic acid could have made polysaccharides negatively charged. This result had an significant influence on the bioactivities of polysaccharides [31]. Besides, the polysaccharide content of IHRPs was between 30% and 35%. The non-sugar components were mainly organic acids, flavonoids, steroids, and glycoside. Meanwhile, the content of each non-sugar component was relatively low.

### 2.7. Antioxidant Activity Study

The ABTS and DPPH free radical scavenging ability have been extensively studied to evaluate the antioxidant activity of polysaccharides [32]. The scavenging capacity of IHRPs against ABTS and DPPH is displayed in Table 5. As exhibited in Table 5, the scavenging rate of DPPH and ABTS both increased with the increase of IHRPs concentration. When the concentration of IHRPs was 1.0 mg/mL, the DPPH scavenging rate was 76.00% while the ABTS scavenging rate was 99.05%. After calculation, the EC_50_ values of ABTS and DPPH were determined as 0.34 and 0.47 mg/mL. To our knowledge, there is no report about the antioxidant activity of IHRPs. The antioxidant activity of the inflorescences from 12 *Cannabis sativa* L. monoecious cultivars were studied [33]. Among them, pineapple had the strongest scavenging activity against DPPH (EC_50_ = 60.00 μg/mL) while Kc-Virtus had the strongest scavenging capacity against ABTS (EC_50_ = 304.77 μg/mL). In terms of the antioxidant activity of cannabidiol-full spectrum oil, it was reported that its DPPH EC_50_ was 158.0 μg/mL [34]. Additionally, the DPPH EC_50_ of CBD was reported to be 0.937 mM [35]. The free radicals scavenging capacity of CBDV was also studied, i.e., the ABTS EC_50_ was 406.3 μM while the DPPH EC_50_ was 3736 μM [36]. The above results indicated that IHRPs showed great antioxidant activity.

### 2.8. Anti-Aging Activity Study

#### 2.8.1. Cell Viability

**Figure 5:**
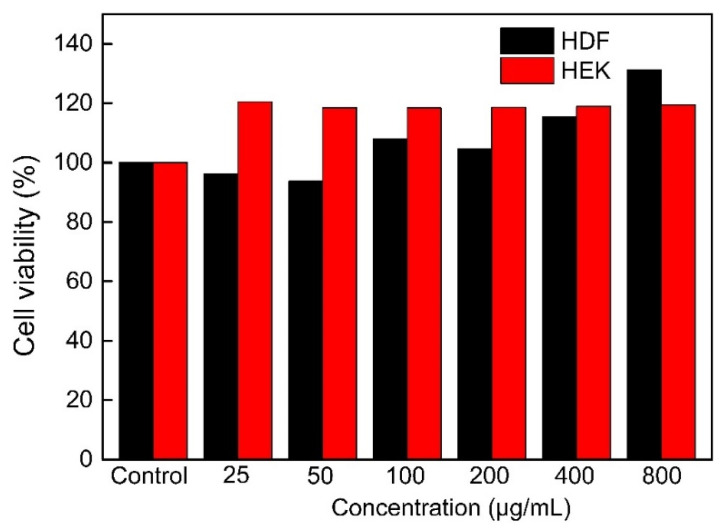
Cell viability of HDF and HEK incubated with IHRPs.

In vitro cytotoxicity experiments are frequently used to assess the toxicity of tested samples [37]. The effects of IHRPs on the cell viability of HDF and HEK, which were the commonly used epidermal cell lines, are displayed in Figure 5. IHRPs almost had no cytotoxicity for HDF and HEK. Furthermore, IHRPs could promote cell proliferation between 100 and 800 μg/mL. To ensure the cell viability was not affected by the concentration of sample, we chose IHRPs concentrations below 400 μg/mL for the following experiments.

#### 2.8.2. Scratch Assay of HDF Cells

**Figure 6:**
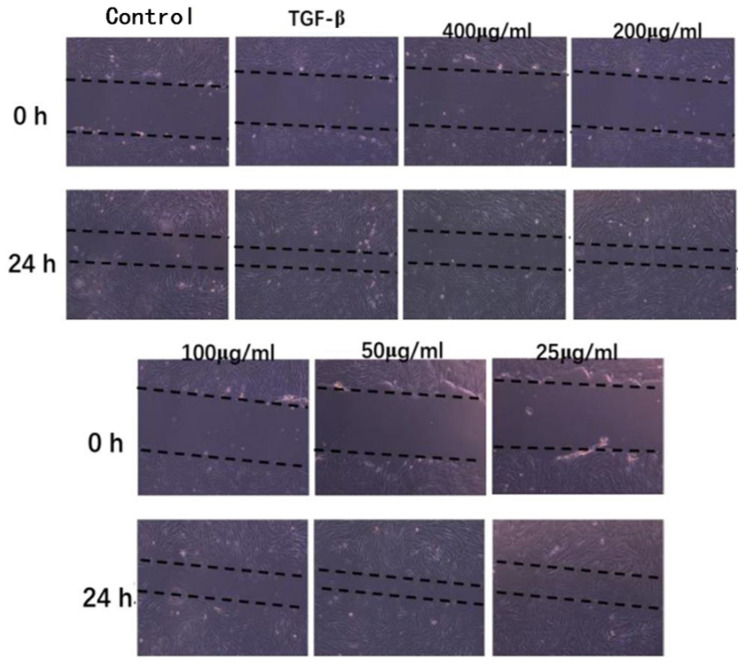
Images of healing condition of HDF scratches after 24 h.

The scratch assay is an in vitro method widely used to evaluate the contribution of cellular and molecular mechanisms to cell proliferation and migration [38]. The images of healing condition of HDF scratches are shown in Figure 6. IHRPs of different concentrations were all beneficial to the healing of cell scratches compared with the control. Moreover, when the concentration of IHRPs was between 50 and 200 μg/mL, IHRPs could significantly promote the healing of cell scratches. The healing rate of HDF scratches after 24 h was 64.51 ± 3.69% (*p* < 0.01), 58.03 ± 3.90% (*p* < 0.05), and 66.21 ± 6.60% (*p* < 0.01) respectively when the IHRPs concentration was 50, 100, and 200 μg/mL, as displayed in Table 6. Besides, the healing effect of IHRPs was very close to that of the positive control TGF-β (62.29 ± 4.69%, *p* < 0.01). The results of scratch assay indicated that IHRPs could obviously accelerate the healing of cell scratches and promote the cell proliferation.

#### 2.8.3. qRT-PCR Analysis of HDF Gene Expression

**Figure 7:**
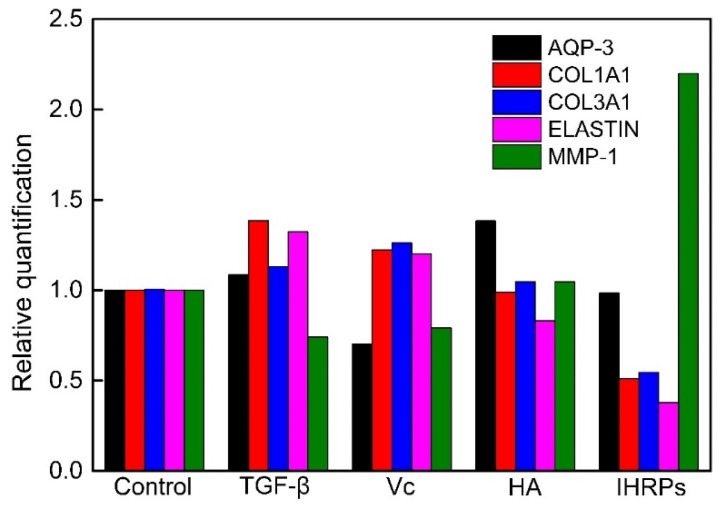
Relative quantification of the anti-aging genes of HDF incubated with IHRPs.

Several studies have been conducted to evaluate the anti-aging potential of different plants [39,40]. The relative quantification of anti-aging genes of HDF is displayed in Figure 7. As shown in the figure, TGF-β, Vc, and HA were used as the positive control. The order of AQP-3 relative quantification was HA > TGF-β > IHRPs > Vc. The order of COL1A1 relative quantification was TGF-β > Vc > HA > IHRPs. The order of COL3A1 relative quantification was Vc > TGF-β > HA > IHRPs. The order of ELASTIN relative quantification was TGF-β > Vc > HA > IHRPs. The order of MMP-1 relative quantification was IHRPs > HA > Vc > TGF-β. Therefore, compared with the positive control, IHRPs nearly had no positive effect on the expression of AQP-3, COL1A1, COL3A1, and ELASTIN. However, IHRPs significantly promoted the expression of MMP-1. MMP-1 is primarily generated by keratinocytes and is mainly used for decomposition and fragmentation of skin collagen fibers [41]. In summary, IHRPs could promote HDF proliferation and the expression of anti-aging related genes, indicating the anti-aging and skin repair potentials of IHRPs.

## 3. Materials and Methods

### 3.1. Materials and Reagents

IHR was provided by Yunnan Hempmon Pharmaceuticals Co., Ltd. (Kunming, China). Phenol, α-naphthol, sulfuric acid, carbazole, Coomassie blue G-250, phosphoric acid, ethanol, chloroform, and monosaccharide control were supplied by Sinopharm Chemical Reagent Co., Ltd. (Shanghai, China). Galacturonic acid, glucuronic acid, guluronic acid, and arbutin were bought from Sigma-Aldrich Co., Ltd. (St. Louis, MO, USA). Human dermal fibroblast (HDF), human epidermal keratinocytes (HEK), and complete cell medium were bought from Sciencell Co., Ltd. (Carlsbad, CA, USA). 12-well plates and 96-well plates were offered by Corning Co., Ltd. (Corning, NY, USA). CCK-8 cell viability assay kit was bought from DOJINDO Biology Co., Ltd. (Tokyo, Japan) and other detection kits were offered by Takara Co., Ltd. (Takara, Japan).

### 3.2. Optimization of IHRPs Extraction

#### 3.2.1. Comparison of Different Extraction Methods

Percolation extraction: 100 g IHR was added into 2000 mL deionized water. The extraction was performed with a flow rate of 150 ± 50 mL/h after 2 h. Heating extraction: 100 g IHR was added into 2000 mL deionized water. The extraction was performed at 98 °C for 1 h and the process was repeated twice. Ultrasonic-assisted extraction: 100 g IHR was dissolved in 2000 mL deionized water. The extraction was conducted for 0.5 h at 60 °C and the process was repeated twice.

The filtrates were concentrated using a vacuum rotary evaporator. Then the solution was dried by a vacuum drier. The polysaccharide content was determined to choose an appropriate method.

#### 3.2.2. Single-Factor Experiments

For the extraction of IHRPs, 100 g IHR was added into a certain amount of deionized water. Extraction temperature (40, 60, 80, and 98 ± 2 °C), solid–liquid ratio (1:6, 1:8, 1:10, and 1:15), extraction time (0.5, 1.0, 1.5, and 2 h), pH (3, 5, 7, 9, and 11), and number of successive extractions (1, 2, 3, 4) were studied separately to evaluate the influence of individual factors on IHRPs extraction.

#### 3.2.3. Orthogonal Experimental Design

Extraction conditions were further optimized by orthogonal experimental design. As shown in Table 7, the four selected variables were extraction temperature (60, 80, and 98 ± 2 °C), R_S/L_ (1:6, 1:8, and 1:10), number of successive extractions (1, 2, and 3), and pH (4, 7, and 10). The orthogonal experiments were divided into 9 groups. The weight of polysaccharides was considered as the target to estimate the extraction conditions.

### 3.3. Screen of IHRPs Alcohol Precipitation Conditions

The extraction solution was obtained by the optimum condition according to 3.2 and 400 mL ethanol was added. The experiments were conducted based on the different rate of adding alcohol, stirring method, and cooling rate in Table 8. The precipitate was obtained after centrifugation and dried. Then the weight and polysaccharide content were acquired to determine the suitable alcohol precipitation conditions.

### 3.4. Determination of Polysaccharide Yield and Chemical Composition of IHRPs

The protein content was calculated based on the Bradford method and the bovine serum albumin (BSA) was employed as a standard [42]. The total sugar content of IHRPs was acquired according to the phenol-sulfate method and the glucose was used as a standard [43]. The uronic acid content was obtained by a carbazole-sulfuric acid method [44]. Polysaccharide yield was obtained by the Equation (1).

(1) $$Y_{P}\;\left(\%\right)=m_{A}/m_{B}*100$$

where *Y_P_* refers to the polysaccharide yield, *m_A_* refers to the weight of IHRPs, and *m_B_* represents the weight of IHR employed for polysaccharide extraction.

### 3.5. Purification of IHRPs

First, 100 g crude IHRPs was prepared by the optimum condition determined in Section 3.2 and Section 3.3. The crude IHRPs were further purified by different methods.

Activated carbon adsorption: 10 g crude IHRPs was added into 200 mL deionized water. The activated carbon of 1%, 2%, 4%, and 8% IHRPs weight was mixed with the solution, respectively. Then, the solution was agitated at 60 °C for 1 h and filtrated. After washing by deionized water, the filtrate was concentrated and dried.

Membrane filtration: 10 g crude IHRPs was added into 500 mL deionized water. Subsequently, the solution was filtrated by membranes with different molecular weight (30,000 Da, 10,000 Da, 1000 Da, and 500 Da) continuously. The filtrate and retentate were both collected and dried. Finally, IHRPs with different molecular weight were obtained.

Sevage method: 1 g crude IHRPs was added into 100 mL deionized water. Then the solution was blended with Sevage reagent (n-butanol: chloroform = 1: 5 (*v*/*v*)). The mixture was stirred for 15 min and transferred to the separatory funnel for stratification. The above process was repeated for 5 times. Subsequently, the organic phase and the aqueous phase were both dried.

Column chromatography: 10 g weakly-basic anion exchange resin was immersed in deionized water for 2 h, then loaded into a glass column (2 × 30 cm). 10 g crude IHRPs was added into 200 mL deionized water. Subsequently, the solution was slowly passed through the resin layer. The column was rinsed with 100 mL 30% ethanol. The eluent was concentrated and dried.

### 3.6. Monosaccharide Composition Analysis

The monosaccharide composition test was performed according to the previous method with slight modifications [45]; 5 mg IHRPs was hydrolyzed with 1 mL trifluoroacetic acid (TFA, 2 M) for 6 h at 105 °C. Subsequently, the solution was dried under nitrogen atmosphere. The dried hydrolysate was added into 5 mL deionized water after TFA was removed by methanol. Afterwards, 0.5 mL of 0.3 M NaOH solution and 1 mL of 0.5 M 1-phenyl-3-methyl-5-pyrazolone (PMP) methanol solution were added into the hydrolysate. The obtained solution was kept in a water bath for 2 h at 70 °C for derivatization. Then, 0.5 mL HCl solution (0.3 M) was added for neutralization. The solution was mixed with 1 mL chloroform for HPLC analysis. Several monosaccharides were used as references. Chromatographic conditions: Mobile phase A (82%): 0.1 M KH_2_PO_4_ solution. Mobile phase B (18%): acetonitrile. Column: C18 (5 μm, 4.6 × 250 mm). Injection volume: 10 μL. Flow rate: 1.0 mL/min. Detection wavelength: 245 nm. Column temperature: 30 °C.

### 3.7. Fourier Transform Infrared Spectroscopy (FT-IR)

The samples for FT-IR analysis were prepared by mixing 5 mg IHRPs with 125 mg KBr. The FT-IR spectra of samples were obtained between 4000 and 500 cm^−1^.

### 3.8. Antioxidant Activity Study

#### 3.8.1. DPPH Radical Scavenging Activity

The DPPH free radical scavenging activity of IHRPs was evaluated based on a reported method with a few modifications [46]. In short, 2 mL sample solution (0.2, 0.4, 0.6, 0.8, and 1.0 mg/mL) was blended with 2 mL DPPH solution (0.1 mM). The mixture was kept for 30 min in the dark, and then the absorbance (Abs) was obtained at 517 nm. The DPPH scavenging rate was estimated with the following equation (Equation (2)) and the 50% effective concentration (EC_50_) was counted.

(2) $$R_{SD}=(1-\frac{A_{i}-A_{j}}{A_{0}})\times 100\%$$

where $R_{SD}$ represents the free radical scavenging rate, $A_{i}$ refers to the Abs of the reaction system (DPPH with the sample), $A_{j}$ is the Abs of the sample background (solvent with the sample); and $A_{0}$ is the Abs of the negative control (DPPH with solvent).

#### 3.8.2. ABTS Radical Scavenging Activity

The ABTS scavenging activity of IHRPs was evaluated based on a previous method [47]. In short, potassium persulfate solution (2.45 mM) was blended with the ABTS solution (7 mM) in dark for 12 h. The obtained ABTS solution was diluted 50 times to the Abs of 0.70 ± 0.02 at 734 nm. Subsequently, 1 mL sample solution (0.2, 0.4, 0.6, 0.8, and 1.0 mg/mL) was added into 4 mL ABTS solution. The mixture was shaken rapidly for 1 min and placed in the dark for 6 min. Then the Abs was measured at 734 nm and Vc was used as a positive control. The ABTS scavenging rate was calculated with Equation (2).

### 3.9. Anti-Aging Activity Study

#### 3.9.1. Cell Culture

Human dermal fibroblast (HDF) and human epidermal keratinocytes (HEK) were purchased from ScienCell Co., Ltd. The cells were cultured with DMEM medium containing 10% FBS. The culture condition was 37 °C with 5% CO_2_.

#### 3.9.2. Cell Viability

Cell Counting Kit-8 (CCK-8) assay was carried out to determine the cytotoxicity of IHRPs against HDF and HEK cells. The cells were cultured in 96-well plates with a concentration of 2 × 10^4^ cells/well for 48 h. Afterwards, 100 μL fresh medium containing samples was added and the wells were incubated for 24 h. Then CCK-8 solution was added to wells according to the instruction of test kits and the plate was incubated for 2 h. The Abs at 450 nm was obtained to calculate the cell viability.

#### 3.9.3. Scratch Assay

HDF was cultured at 37 °C with 5% CO_2_. Then the cells were seeded into a 12-well plate at a concentration of 1 × 10^5^ cells/well. After incubated for 48 h, the scratch was created on the HDF cellular layer by the syringe needle. The cell fragments were cleaned by PBS and the samples with different concentrations were added. Subsequently, the healing of cell layer scratches was observed after 24 h to evaluate the effect of IHRPs on proliferation of HDF. The obtained images were quantified by image J software [48]. Transforming growth factor-β (TGF-β) was used as the positive control. Healing rate was calculated according to Equation (3).

(3) $$Healing\;rate\;\left(\%\right)=\left(A_{1}-A_{2}\right)/A_{1}*100$$

where *A*_1_ represents the initial scratch area and *A*_2_ refers to the final scratch area.

#### 3.9.4. Quantitative RT-PCR (qRT-PCR) Analysis

Then the cells were seeded into 12-well plates with the concentration of 1 × 10^5^ cells/well. After incubated for 48 h, the samples were mixed and cultured for another 24 h. Total RNA was extracted and cDNA was synthesized. The qRT-PCR analyses for aquaporin gene (AQP-3), collagen gene (COL1A1 and COL3A1), elastin gene (ELASTIN), and matrix metalloproteinase (MMP-1) were performed using a real-time PCR system (Applied Biosystems Life Technologies, Inc., ABI StepOnePlus). Relative quantification was conducted with the comparative CT method (2^-ΔΔCt^ method). Hyaluronic acid (HA), Vc, and TGF-β were employed as the positive control.

### 3.10. Statistical Analysis

Results were displayed as the mean ± SD (*n* = 3). Statistical significance was performed by ANOVA. Values of *p* < 0.05 were considered to be statistically significant.

## 4. Conclusions

In this work, the polysaccharide extraction from IHR was optimized by single-factor experiments and orthogonal experimental design. The optimum heating extraction conditions were extraction temperature was 98 °C, solid–liquid ratio 1:10, extraction time 1 h, number of successive extractions 2, and pH 4. The extraction ratio and the polysaccharide content were 20.12% and 12.35% respectively at the conditions. Additionally, the suitable alcohol precipitation conditions were pumping with 2 L/h, stirring continuously, and ice-water bath for 4 h. The crude IHRPs was further purified by column chromatography and the polysaccharide/protein contents of purified IHRPs were 34.44% and 1.61%. The monosaccharide composition of IHRPs was: fucose (1.33%), arabinose (19.60%), rhamnose (10.41%), galactose (20.87%), glucose (27.42%), xylose (4.23%), ribose (3.12%), galacturonic acid (6.22%), guluronic acid (0.28%), and glucuronic acid (2.37%). The FT-IR demonstrated the polysaccharide skeleton of IHRPs. Besides, the EC_50_ values of ABTS and DPPH radicals were 0.34 and 0.47 mg/mL, showing the great antioxidant activity of IHRPs. IHRPs also could promote the cell proliferation of HDF and HEK and healing of cell scratches. Moreover, IHRPs could significantly promote the expression of MMP-1. Therefore, it is believed that the polysaccharides from industrial hemp residues could be developed as potential antioxidant and anti-aging products for cosmetics or functional foods.

## Figures and Tables

**Table 1:** Orthogonal experiments under different extraction conditions.

Groups	Temperature/°C	Solid–Liquid Ratio	Number of Successive Extractions	pH	Extract Weight/g	Polysaccharide Content/%	Actual Polysaccharide Weight/g
1	60	1:6	1	4	6.51	8.42	0.55
2	60	1:8	2	7	9.1	7.66	0.70
3	60	1:10	3	10	11.28	6.80	0.77
4	80	1:6	2	10	13.87	11.85	1.64
5	80	1:8	3	4	16.68	12.79	2.13
6	80	1:10	1	7	13.13	9.21	1.21
7	98	1:6	3	7	17.95	12.17	2.18
8	98	1:8	1	10	16.86	11.57	1.95
9	98	1:10	2	4	20.07	12.26	2.46
Average 1	0.67	1.46	1.24	1.71			
Average 2	1.66	1.59	1.60	1.36			
Average 3	2.20	1.48	1.69	1.45			
Range	1.52	0.14	0.46	0.35			

**Table 2:** Validation experiments of extraction conditions of group 5 and 9.

Groups	Temperature/°C	Solid–Liquid Ratio	Number of Successive Extractions	pH	Extraction Ratio/%	Polysaccharide Content/%
5	80	1:8	3	4	20.38 ± 0.19	12.21 ± 0.16
9	98	1:10	2	4	20.12 ± 0.55	12.35 ± 0.26

**Table 3:** Effects of different alcohol precipitation conditions.

Groups	Rate of Adding Alcohol	Stirring Method	Cooling Rate	Precipitate Weight/g	Polysaccharide Content/%
1	One-time addition	Stirring for 0.5 h	Ice-water bath for 4 h	0.70	19.83
2	One-time addition	Stirring continuously	Cooling to 0 °C with 20 °C/h and incubation for 4 h	0.58	21.58
3	Pumping with 2 L/h	Stirring for 0.5 h	Cooling to 0 °C with 20 °C/h and incubation for 4 h	0.63	23.75
4	Pumping with 2 L/h	Stirring continuously	Ice-water bath for 4 h	0.62	25.28

**Table 4:** Polysaccharide/protein contents of IHRPs purified by column chromatography.

Processes	IHR/g	Resin/g	IHRPs/g	*Y_P_*/%	Polysaccharide Content/%	Protein Content/%
Purification after heating extraction	200	20	15.26	2.35	30.74	1.79
Purification after alcohol precipitation	200	20	12.74	2.19	34.44	1.61

**Table 5:** Antioxidant activity of IHRPs.

IHRPs Concentration (mg/mL)	0.2	0.4	0.6	0.8	1.0
DPPH *R_SD_* (%)	25.87	44.26	58.44	69.07	76.00
ABTS *R_SD_* (%)	32.73	58.26	74.49	90.22	99.05

**Table 6:** Healing rate of HDF scratches (* *p* < 0.05, ** *p* < 0.01).

Samples	Concentration (μg/mL)	Healing Rate (%)
Control	/	45.13 ± 5.31
TGF-β	0.02	62.29 ± 4.69 **
IHRPs	400	49.21 ± 5.91
	200	66.21± 6.60 **
	100	58.03 ± 3.90 *
	50	64.51 ± 3.69 **
	25	52.22 ± 5.64

**Table 7:** Orthogonal design table of extraction condition.

Levels	Temperature	R_S/L_	Number of Successive Extractions	pH
1	60 °C	6	1	4
2	80 °C	8	2	7
3	98 ± 2 °C	10	3	10

**Table 8:** Screening of alcohol precipitation conditions.

Groups	Rate of Adding Alcohol	Stirring Method	Cooling Rate
1	One-time addition	Stirring for 0.5 h	Ice-water bath for 4 h
2	One-time addition	Stirring continuously	Cooling to 0 °C at a speed of 20 °C/h and incubation for 4 h
3	Pumping with 2 L/h	Stirring for 0.5 h	Cooling to 0 °C at a speed of 20 °C/h and incubation for 4 h
4	Pumping with 2 L/h	Stirring continuously	Ice-water bath for 4 h

## Data Availability

Not applicable.

## Sample Availability

Samples of the compounds IHR and IHRPs are available from the authors.
